# Mapping Antimicrobial Stewardship in Undergraduate Medical, Dental, Pharmacy, Nursing and Veterinary Education in the United Kingdom

**DOI:** 10.1371/journal.pone.0150056

**Published:** 2016-02-29

**Authors:** Enrique Castro-Sánchez, Lydia N. Drumright, Myriam Gharbi, Susan Farrell, Alison H. Holmes

**Affiliations:** 1 NIHR Health Protection Research Unit in Healthcare Associated Infection and Antimicrobial Resistance at Imperial College London, London, United Kingdom; 2 Department of Medicine, University of Cambridge, Cambridge, United Kingdom; 3 Department of Surgery & Cancer, Imperial College London, London, United Kingdom; University at Buffalo, SUNY, UNITED STATES

## Abstract

**Objectives:**

To investigate the teaching of antimicrobial stewardship (AS) in undergraduate healthcare educational degree programmes in the United Kingdom (UK).

**Participants and Methods:**

Cross-sectional survey of undergraduate programmes in human and veterinary medicine, dentistry, pharmacy and nursing in the UK. The main outcome measures included prevalence of AS teaching; stewardship principles taught; estimated hours apportioned; mode of content delivery and teaching strategies; evaluation methodologies; and frequency of multidisciplinary learning.

**Results:**

80% (112/140) of programmes responded adequately. The majority of programmes teach AS principles (88/109, 80.7%). ‘Adopting necessary infection prevention and control precautions’ was the most frequently taught principle (83/88, 94.3%), followed by 'timely collection of microbiological samples for microscopy, culture and sensitivity’ (73/88, 82.9%) and ‘minimisation of unnecessary antimicrobial prescribing’ (72/88, 81.8%). The ‘use of intravenous administration only to patients who are severely ill, or unable to tolerate oral treatment’ was reported in ~50% of courses. Only 32/88 (36.3%) programmes included all recommended principles.

**Discussion:**

Antimicrobial stewardship principles are included in most undergraduate healthcare and veterinary degree programmes in the UK. However, future professionals responsible for using antimicrobials receive disparate education. Education may be boosted by standardisation and strengthening of less frequently discussed principles.

## Introduction

The threat posed by antimicrobial resistance has been equated to climate change [1], with the inappropriate use of antimicrobials in human and animal health resulting in resistant organisms, which in turn create, at a minimum, unresolved challenges such as increased health care utilisation and costs [2], and at the extreme, excess morbidity and mortality [3]. The contributions to antimicrobial resistance are multidimensional and range from behavioural dynamics of healthcare workers to the biology of the microorganisms [4], and thus multimodal interventions have been suggested to be of most benefit. Antimicrobial stewardship (AS), an integrated and multidisciplinary approach that includes the selection of appropriate drugs, enhanced surveillance of prescribing and use, implementation of prescribing guidelines and policies, inclusion of infection prevention and control strategies, and increased efforts on audit and education, has been promoted to arrest the rise of antimicrobial resistance [5].

Despite considerations of education as a fundamental tool to combat antimicrobial resistance [6], some studies have identified gaps in the provision of skills, knowledge and attitudes related to antimicrobials necessary to deliver effective and safe care [7], therefore suggesting an unclear understanding of what is contributing to these gaps in skills and knowledge. Furthermore, such gaps appear to be coupled with reports of undergraduate students demonstrating an interest in receiving increased antimicrobial education during their degree programmes [8,9], suggesting that not enough emphasis is placed on antimicrobial prescribing and stewardship in healthcare educational degree programmes.

To date, however, there has been limited investigation into human and animal health students' knowledge and perceptions of antimicrobial stewardship, and no study to our knowledge has explored the stewardship education delivered by universities. We intended therefore to describe the antimicrobial stewardship learning delivered in undergraduate curricula across disciplines involved in prescribing, administering or reviewing antimicrobials (medicine, nursing, pharmacy, dentistry and veterinary medicine) in the United Kingdom (UK). We aimed to explore how key antimicrobial stewardship principles were delivered, the pedagogies utilised, and the background of those providing education, with a view to identify areas for improvement and aid future capacity building initiatives in education.

## Participants and Methods

### Design and setting

We conducted a cross-sectional survey exploring recommended antimicrobial stewardship principles (S1 Box) included in the curriculum of undergraduate medicine, pharmacy, nursing, dentistry and veterinary medicine university courses in the UK. The selected principles reflect key dimensions in current antimicrobial stewardship policy in the UK [10].

### Participants and recruitment

We identified universities from the official Universities and Colleges Admissions Service (UCAS) list in 2013. Course organisers or module leaders of university programmes offering undergraduate degrees in human and veterinary medicine, dentistry, pharmacy and nursing were identified via the information available on universities’ web pages and invited to complete an electronic survey on Google Forms. The survey was available online for a period of 8 weeks between March and May 2013. Participation was voluntary and the responses were anonymous. No incentives were offered.

### Data collection

A team of investigators including an academic research nurse and an educational manager developed the survey instrument (S1 Appendix). We collected information on university and degree programme characteristics; presence of antimicrobial stewardship in the course curriculum; principles of stewardship taught; estimate of number of hours apportioned to antimicrobial stewardship; professional background of lecturers in stewardship sessions; mode of stewardship content delivery (face to face, online or mixture of both); teaching strategies employed in antimicrobial stewardship sessions (lectures, case studies, student presentations, activities in clinical settings, use of simulators or other virtual environments, etc.); methodologies used to evaluate students, as well as arrangements for multidisciplinary learning for some or all of the content. The survey content and the usability of the electronic platform was piloted amongst hospital doctors, nurses and pharmacists as well as MSc Infection students that included nurses and pharmacists. Participants were able to review their options as all questions appeared on one single page.

As secondary source of data, in July 2013 we submitted a request to each university under the provisions of the UK Freedom of Information Act (2000). As public bodies, universities are required to submit a response within 20 working days of the request. However, the stipulations included in the Act allow universities to refuse the request for information on grounds of cost or business sensitivity.

### Statistical analysis

Descriptive analysis was conducted. Analyses included cross tabulations and tests of central tendency and dispersion. Associations between disciplines and antimicrobial stewardship components, teaching and evaluation were explored. Differences in antimicrobial stewardship hours between different disciplines were compared using parametric or nonparametric tests, depending on the normality of distributions. Statistical analysis was performed with STATA v10.1 (STATA Corp, College Station, TX).

### Ethical approval

The study obtained approval by Imperial College Research Ethics Committee. All participants received an information sheet with information about the research team, the purpose of the study, the survey completion time, and the confidential and anonymous nature of responses. We considered that submission of responses via the electronic form indicated agreement to participate in the survey.

## Results

We contacted 140 UK undergraduate programmes in medicine, nursing, veterinary medicine, dentistry or pharmacy. Of these, 112 (80%) submitted a response, but only 109 (77.85%) were sufficiently completed to be analysed (three universities provided a generic response or marketing brochures). Table 1 presents response rates for each discipline.

**Table 1:** Number of participating courses, by discipline.

Discipline	Response rate n/N (%)
Dentistry	16/16 (100)
Medicine	24/34 (70.5)
Nursing	51/58 (87.9)
Pharmacy	15/26 (57.6)
Veterinary medicine	6/6 (100)
**Total**	112/140 (80)

### Courses including AS in their curriculum

Of the 109 courses submitting adequately completed responses, 88 (80.7%, 95% Confidence Interval [CI] 73.29–88.10) reported explicitly teaching AS in their curriculum. However, there was some variation in frequency amongst disciplines. All dentistry and veterinary medicine schools reported teaching AS, with similar percentages in medical (23/24, 95.8%, 95% CI 87.77–100) and pharmacy schools (13/15, 86.6%, 95% CI 69.36–100). On the contrary, only 31/49 (63.2%, 95% CI 49.69–76.70) nursing schools incorporated any teaching about stewardship.

### Teaching of recommended AS principles

**Fig 1:**
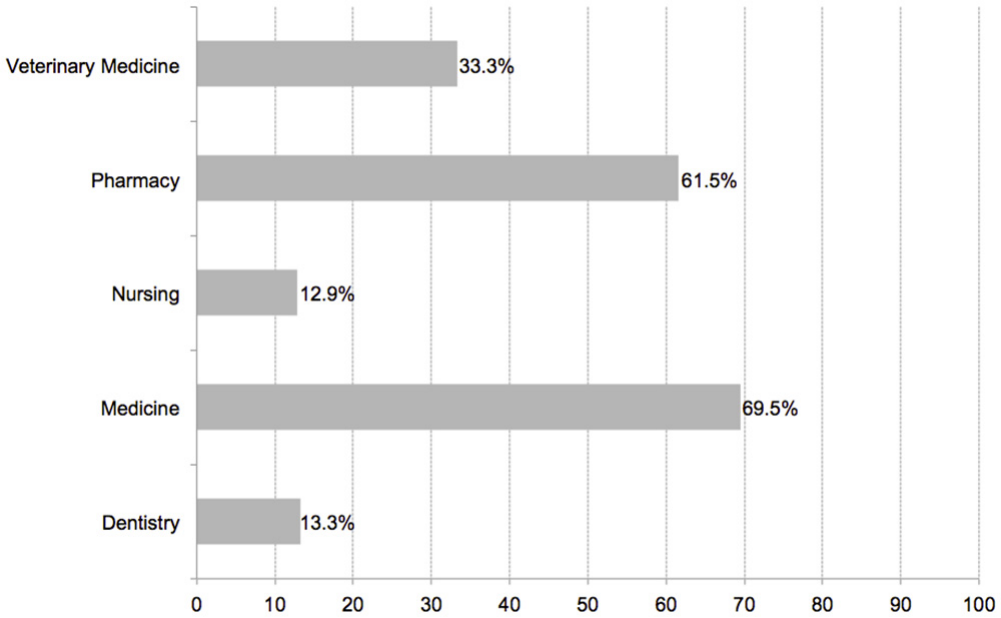
Faculties teaching all recommended AS principles, by discipline.

Whilst 88 courses stated the inclusion of antimicrobial stewardship in their curricula, only 32/88 (36.3%, 95% CI 26.25–46.34) included all recommended principles. The differences between disciplines were notable, ranging from 4/31 (12.9%, 95% CI 11–24.69) nursing schools to 16/23 (69.5%, 95% CI 50.68–88.31) medical schools. Fig 1 presents the results for all disciplines.

Table 2 displays the frequency of teaching for each principle recommended in antimicrobial stewardship guidelines, presented by discipline. The most frequently cited principle (83/88, 94.3%, 95% CI 89.45–99.14) was ‘adopting necessary infection prevention and control precautions’, followed by 'timely collection of microbiological samples for microscopy, culture and sensitivity’ (73/88, 82.9%, 95% CI 75.03–90.76) and ‘minimisation of unnecessary antimicrobial prescribing’ (72/88, 81.8%, 95% CI 73.73–89.86). In contrast, the ‘use of intravenous administration only to patients who are severely ill, unable to tolerate oral treatment, or where oral therapy would not provide adequate tissue penetration’ was reported in ~50% of courses. These percentages were maintained even if only responses from human health courses (medicine, nursing, pharmacy and dentistry) were considered.

**Table 2:** Antimicrobial stewardship principles included in undergraduate education programmes, by discipline.

Antimicrobial stewardship principle	Dentistry n/N (%)	Medicine n/N (%)	Nursing n/N (%)	Pharmacy n/N (%)	Veterinary Medicine n/N (%)	Total n/N(%)
Minimisation of unnecessary prescribing of antimicrobials	13/13 (100)	21/22 (95.4)	19/33 (57.5)	14/14 (100)	5/6 (83.3)	72/88 (81.8)
Timing of antimicrobial administration	13/13 (100)	21/22 (95.4)	19/33 (57.5)	14/14 (100)	5/6 (83.3)	72/88 (81.8)
Therapeutic drug monitoring	2/12 (16.6)	20/22 (90.9)	13/32 (40.6)	11/14 (78.5)	4/6 (66.6)	50/86 (58.1)
Need for standard infection prevention and control precautions	13/13 (100)	22/22 (100)	32/33 (96.9)	12/14 (85.7)	5/6 (83.3)	83/88 (94.3)
Collection of appropriate specimens for microscopy, culture and sensitivity	9/13 (69.2)	21/22 (95.4)	26/33 (78.7)	12/14 (85.7)	5/6 (83.3)	73/88 (82.9)
Intravenous use only in severely ill patients, unable to tolerate oral treatment, or where oral treatment would not guarantee coverage or tissue penetration	7/13 (53.8)	18/22 (81.8)	14/32 (43.7)	10/13 (76.9)	4/6 (66.6)	53/86 (61.6)
Review microbiology results daily and de-escalate to pathogen-directed narrow-spectrum treatment promptly	4/13 (30.7)	18/22 (81.8)	10/32 (31.2)	11/14 (78.5)	4/6 (66.6)	47/87 (54.0)
Review need for intravenous treatment daily and switch to oral route promptly	3/13 (23.0)	18/22 (81.8)	9/32 (28.1)	10/14 (71.4)	4/6 (66.6)	44/87 (50.5)
Require single dose surgical prophylaxis regimens as appropriate	5/13 (38.4)	16/22 (72.7)	9/32 (28.1)	9/14 (64.2)	4/6 (66.6)	43/87 (49.4)

### Background of teachers delivering AS content

We explored the profession of teachers in the 88 courses reporting antimicrobial stewardship in their curriculum, with the view that exposing students to multidisciplinary instructors may positively influence their antimicrobial clinical teamwork. Overall, 55/88 courses (62.5%, 95% CI 52.38–72.61) included tutors with professional backgrounds different to the students they were teaching to (for instance, 13/15 (86.6%) dentist courses employed antimicrobial stewardship lecturers with backgrounds other than dentistry. In medicine 19/23 (82.6%) of courses employed antimicrobial lecturers with backgrounds other than medicine. The results for the rest of disciplines were nursing 21/31 (67.7%), pharmacy 8/13 (61.5%), and veterinary medicine 3/6 (50%).

### Mode of AS content delivery

The delivery of stewardship teaching included a mixture of face-to-face and online pedagogies. However, there was heterogeneity amongst the disciplines in the uptake of online platforms and the use of blended learning (combining face-to-face and online methods). Nursing (22/30, 73.3%, 95% CI 57.46–89.13) reported the highest use of blended approaches, whilst the majority of pharmacy (11/13, 84.6%, 95% CI 64.97–100) and dentistry courses (11/15, 73.3%, 95% CI 50.91–95.68) were delivered face-to-face. Around 50% (12/23, 52.1%, 95% CI 31.68–72.51) of medical courses reported the use of face-to-face methods.

### AS teaching methodologies

The variety of teaching strategies reported by is presented in Table 3. In nine cases (10.2%), lectures would be the only teaching methodology employed.

**Table 3:** Antimicrobial stewardship teaching methodologies used in undergraduate education programmes, by discipline.

	Dentistry (n = 15)	Vet Med (n = 6)	Pharmacy (n = 14)	Medicine (n = 24)	Nursing (n = 29)	Total(n = 88)
Lecture	4	0	2	1	2	9
Lecture + case studies	4	0	2	3	1	10
Lecture + case studies + other	0	0	5	2	3	10
Lecture + clinical setting	1	0	0	0	1	2
Lecture + clinical setting + other	0	1	0	0	5	6
Lecture + case studies + clinical setting	3	0	1	2	3	9
Lectures + case studies + clinical setting + other	2	3	3	13	8	29
Other methodologies/ combined methodologies	1	2	1	3	6	13

Clinical setting = activities in clinical setting; Other includes use of simulators, problem-based learning activity, student presentations, reflective practice journals.

### AS evaluation approaches

The diversity of teaching methodologies was mirrored by the multiple evaluation approaches reported by the universities, and is presented in Table 4. The use of objective structured examinations, alone or in combination with short-answer questions and essays, was the preferred practice. One of the institutions reported no formal assessment or evaluation for students in the area of antimicrobial stewardship.

**Table 4:** Antimicrobial stewardship evaluation methodologies used in undergraduate programmes, by discipline.

	Dentistry (n = 15)	Vet Med (n = 6)	Pharmac (n = 14)	Medicine (n = 24)	Nursing (n = 26)	Total per approach
OSCE	0	0	0	0	4	4
Written exam	0	0	0	2	2	4
No formal assessment	0	0	0	0	1	1
Essay	0	0	0	0	1	1
Clinical assessment	0	0	0	0	1	1
Essay + OSCE	1	0	0	2	1	4
Essay + written exam	2	1	1	3	3	10
Essay + OSCE + other	3	1	4	8	4	20
Essay + clinical assessment + other	0	0	0	0	2	2
OSCE + written exam	6	2	4	6	2	7
OSCE + presentation + other	1	0	2	2	3	5
OSCE + portfolio + other	0	0	1	1	0	2
Other grouped categories	2	3	2	0	4	11

Other includes any of the following, single or in combination: Objective structured clinical examination (OSCE); multiple choice question; short assessment; long assessment; single best answer; student presentation.

### Availability of multidisciplinary learning opportunities

18/88 (20.4%, 95% CI 11.98–28.81) of courses with antimicrobial stewardship in the curriculum facilitated information about the availability of multidisciplinary learning opportunities. Pharmacy schools (4/13, 30.7%, 95% CI 5.62–55.77) were more likely to include opportunities were some or all of the content was learned together with students from other disciplines than medical (6/23, 26.1%, 95% CI 8.15–44.04) dental (3/15, 20%, 95% CI 0–40.24) schools, veterinary medicine (1/6, 16.6%, 95% CI 0–46.37) or nursing schools (4/31, 12.9%, 95% CI 1.10–24.69).

### Hours of AS included in curriculum, by discipline

Of 88 courses reporting AS in their curricula, 69 (78.4%, 95% CI 69.80–86.99) provided the number of hours. The median number of hours for all courses was 10 (interquartile range 3–100), with 17.75 (interquartile rage 8.87–42.62) hours in medical schools (n = 13); veterinary medicine 15.5 (interquartile rage 12.25–40.87) hours (n = 4); pharmacy 12 (interquartile rage 7–25) hours (n = 12); nursing 10 (interquartile rage 4.5–13.5) hours (n = 27); and dentistry 8.5 (interquartile rage 5–10) hours (n = 13) (Kruskal-Wallis H test χ2(4) = 9.165, p = 0.0571).

## Discussion

Our nationwide survey suggests that antimicrobial stewardship is included in the majority of undergraduate medicine, pharmacy, nursing, dentistry and veterinary medicine courses in the UK. However, there are marked differences in the elements of stewardship included within the curricula of the different programmes. Practical elements such as obtaining suitable microbiological samples and the use of adequate infection prevention and control measures appear to take precedence over steps involving executive decision-making, such as reviewing broad-spectrum antibiotic agents once microbiological results are reported back, or considering oral instead of IV administration route. Amongst the different disciplines, nursing faculties presented the most varied profile with ~63% of schools teaching AS but less than 13% including all the recommended steps of stewardship; such results may be attributed to perspectives about the participation of nursing from AS activities [11].

Whilst other studies have measured different aspects of undergraduate students’ understanding, attitudes and behaviours regarding antimicrobials, antimicrobial prescribing or antimicrobial resistance, to our knowledge this is the first published study exploring antimicrobial stewardship as provided by universities in the UK. Additionally, we are the first investigators collecting information from all the main disciplines that prescribe, manage and/or administer antimicrobials, thus providing a richness and comprehensiveness not considered by other surveys that have focused on medical doctors [12–15] or pharmacists [16] exclusively, or from European perspectives that, whilst useful, may be unable to provide detailed information about local contexts [17]. Our findings replicate the variations in exposure to recommended principles and concepts experienced by the students in those studies, including the poor coverage of major principles (e.g. reassessment and duration of antibiotic therapy), as well as observing wide variations in exposure for student within similar educational programmes.

Our use of standardised principles recommended in current UK policy and with extensive common elements with other international antimicrobial stewardship guidelines would ensure that results could be directly compared with future investigations, appearing at a crucial time when national antimicrobial stewardship competencies have been developed [18]. The inclusion of an item referred to adequate infection prevention and control precautions, not strictly an antimicrobial stewardship principle, recognised the close relation of such precautions with the safe collection of microbiological samples.

Our study, however, presents limitations. We did not approach midwifery programmes in our study. We recognize that antimicrobial stewardship knowledge may be delivered in separate sessions across different modules; however, we suggest that if stewardship is to be approached as a cohesive and coherent ‘bundle’ of optimal prescribing behaviours, it would be more appropriate to provide all steps together. In addition, our study identified only the antimicrobial stewardship education provided by universities through selected but broad antimicrobial components, which may have not allowed us to study with accuracy the extent of stewardship teaching. Equally, students may be likely to acquire additional knowledge during clinical placements.

Future professionals responsible for using and managing antimicrobials receive disparate education about stewardship. The impact of education initiatives and continued professional development for graduates in this area may be boosted by curricula standardisation and a focus on strengthening the education about components such as single-dose surgical antibiotic prophylaxis, which seem to be less frequently discussed, using multimodal approaches [19, 20]. Focusing on these components is likely to improve suboptimal clinical practice. The variability in hours and content delivered in UK universities merits further exploration, with consideration towards the technical skills and knowledge involved in stewardship, as well as expertise in communication, negotiation and decision-making. Clearly, applying our selection of antimicrobial stewardship principles, not tailored to each discipline, might explain the variation of the prevalence of teaching of all principles between disciplines. Future studies exploring associations between the educational content provided by universities and the performance or confidence of students in their antimicrobial stewardship skills and knowledge could be of benefit to clarify most favourable approaches. Determining the optimal number of hours to be devoted to antimicrobial stewardship may prove to be problematic, though, due to requirements to incorporate subjective and objective elements, but may be aided by curriculum standardisation efforts. Finally in this regard, our study describes hours of academic learning and does not take into account the educational content that may be acquired by students on clinical placements.

Prescribing and management of antimicrobials appears to be mediated by social interactions between different professions [21,22], with multidisciplinary teamwork practice and training influencing optimal patient outcomes [23]. A proportion of universities in our study offered combined multidisciplinary learning; however, implementing complex educational programmes across disciplines may present logistical difficulties and be unfeasible for all institutions. The frequent use of blended learning platforms highlighted in our study amongst undergraduate institutions may resolve those logistical difficulties and lend sustenance to the development and evaluation of electronic, computerised and/or smartphone-based applications and clinical decision support tools [24].

Whilst antimicrobial stewardship is incorporated explicitly in most human health and veterinary undergraduate courses in the United Kingdom, educators may strengthen the concept by adopting a comprehensive approach with standardised content that emphasises stewardship as a bundle of optimal antimicrobial prescribing behaviours.

However, a majority of programmes do not touch upon all the recommended antimicrobial stewardship principles. In addition, the hours devoted by each discipline to stewardship, together with the teaching and evaluation approaches vary widely and suggest a lack of standardisation in this area.

## Supporting Information

S1 Appendix Questionnaire submitted to universities. (DOCX)

S1 Box Recommended antimicrobial stewardship principles. (DOCX)

S1 CHERRIE Checklist CHERRIE checklist. (DOCX)

S1 File Responses submitted by universities. Only publicly available responses included here.
(CSV)
