# Supplementary material for: Mapping Antimicrobial Stewardship in Undergraduate Medical, Dental, Pharmacy, Nursing and Veterinary Education in the United Kingdom
Source: PLoS One. 2016 Feb 29;11(2):e0150056. doi: 10.1371/journal.pone.0150056 (PMC4771156; doi:10.1371/journal.pone.0150056)
Supplement: S1 Appendix — (DOCX) [file pone.0150056.s001.docx]

**S1 Appendix. Questionnaire submitted to universities**

**1) Was antimicrobial stewardship included in the undergraduate [discipline] curriculum offered by your university in 2012-3?**

**2) What elements of antimicrobial stewardship were included in the undergraduate [discipline] curriculum offered by your university in 2012-3?** (i.e., minimisation of unnecessary prescribing of antimicrobials; timing of antimicrobial administration; therapeutic drug monitoring; need for standard infection prevention and control precautions; collection of appropriate specimens for microscopy, culture and sensitivity; intravenous use only in severely ill patients, unable to tolerate oral treatment, or where oral treatment would not guarantee coverage or penetration; review microbiology results daily and de-escalate to pathogen-directed narrow-spectrum treatment promptly; review need for intravenous treatment daily and switch to oral route promptly; require single dose surgical prophylaxis regimens as appropriate

**3) In the undergraduate [discipline] curriculum offered by your university in 2012-3, how many hours were assigned to antimicrobial stewardship activities?**

**4) What was the background of the lecturers delivering antimicrobial stewardship activities in 2012-3?** (i.e., physician/surgeon; pharmacist; nurse; veterinary doctor; other)

**5) What was the main mode of antimicrobial stewardship content delivery in 2012-3?** (i.e., online teaching; blended teaching (classroom and online activities); face-to-face, classroom-based teaching)

**6) What teaching strategies were used to deliver the antimicrobial stewardship contents?** (i.e., lectures; case studies; student presentations; activities in clinical settings; problem-based teaching; use of simulators or other virtual environments; other)

**7) What method was used to evaluate learners' knowledge about antimicrobial stewardship content?** (i.e., essay; objective structured clinical examination (OSCE) stations; student presentations; student portfolio; short-answer examination; long-answer examination; multiple-choice question examination; other)

**8) Did undergraduates from different professions learn any part of the antimicrobial stewardship content together?**
