# Supplementary material for: Mapping Antimicrobial Stewardship in Undergraduate Medical, Dental, Pharmacy, Nursing and Veterinary Education in the United Kingdom
Source: PLoS One. 2016 Feb 29;11(2):e0150056. doi: 10.1371/journal.pone.0150056 (PMC4771156; doi:10.1371/journal.pone.0150056)
Supplement: S1 Box — (DOCX) [file pone.0150056.s002.docx]

**S1 Box. Recommended antimicrobial stewardship principles**

- Minimisation of unnecessary prescribing of antimicrobials;
- Timing of antimicrobial administration;
- Therapeutic drug monitoring;
- Need for standard infection prevention and control precautions;
- Collection of appropriate specimens for microscopy, culture and sensitivity;
- Intravenous use only in severely ill patients, unable to tolerate oral treatment, or where oral treatment would not guarantee coverage or tissue penetration;
- Review microbiology results daily and de-escalate to pathogen-directed narrow-spectrum treatment promptly;
- Review need for intravenous treatment daily and switch to oral route promptly;
- Require single dose surgical prophylaxis regimens as appropriate.
