# Supplementary material for: Mapping Antimicrobial Stewardship in Undergraduate Medical, Dental, Pharmacy, Nursing and Veterinary Education in the United Kingdom
Source: PLoS One. 2016 Feb 29;11(2):e0150056. doi: 10.1371/journal.pone.0150056 (PMC4771156; doi:10.1371/journal.pone.0150056)
Supplement: S1 CHERRIE Checklist — (DOCX) [file pone.0150056.s003.docx]

**S1 Checklist. CHERRIE checklist**

| **Item Category** | **Checklist Item** |
| --- | --- |
| **Design** | Lines 84, 91, 92 |
| **IRB (Institutional Review Board) approval and informed consent process** | IRB approval (Line 129)  Informed consent (Lines 132-134)  Data protection |
| **Development and pre-testing** | Lines 110-112 |
| **Recruitment process and description of the sample having access to the questionnaire** | Open survey vs closed survey (Lines 92-96)  Advertising the survey We did not advertise the survey but included the link in the invitation email to the selected individuals |
| **Survey administration** | Survey administration (Line 95)  Context (Line 95)  Mandatory/voluntary (Line 97)  Incentives (Line 96)  Time/Date (Line 97)  Randomization of items or questionnaires (Not appropriate)  Adaptive questioning We only had 8 items and no adaptative questioning  Number of Items We had 8 items  Number of screens (pages) The questionnaire had 1 page only  Completeness check We included ‘not applicable’ as appropriate  Review step (Lines 113-114) |
| **Response rates** | Unique site visitor Only selected individuals had access to the link  View rate Only selected individuals had access to the link |
|  | Participation rate (Ratio of unique visitors who agreed to participate/unique first survey page visitors) |
|  | Completion rate (Ratio of users who finished the survey/users who agreed to participate) |
| **Preventing multiple entries from the same individual** | Cookies used We did not use cookies |
|  | IP check: We did not identify or stored any IP  Registration We did not provide login/registration mechanisms |
| **Analysis** | Analysis We only analyzed completed questionnaires. We did not measure time completion  Statistical correction We did not carry out any statistical adjustment, as it was a survey for purposive sample of individuals |
